# Differential expression analysis of *Trichoderma virens* RNA reveals a dynamic transcriptome during colonization of *Zea mays* roots

**DOI:** 10.1186/s12864-019-5651-z

**Published:** 2019-04-11

**Authors:** Elizabeth A. Malinich, Ken Wang, Prasun K. Mukherjee, Michael Kolomiets, Charles M. Kenerley

**Affiliations:** Department of Plant Pathology and Microbiology, Texas A&M University, College Station, TX USA

**Keywords:** *Trichoderma virens*, *Zea mays*, Root colonization, Transcriptome, Phytohormone, Cell wall degrading enzymes, Secondary metabolites, RNA-seq, Differential expression

## Abstract

**Background:**

*Trichoderma* spp. are majorly composed of plant-beneficial symbionts widely used in agriculture as bio-control agents. Studying the mechanisms behind *Trichoderma*-derived plant benefits has yielded tangible bio-industrial products. To better take advantage of this fungal-plant symbiosis it is necessary to obtain detailed knowledge of which genes *Trichoderma* utilizes during interaction with its plant host. In this study, we explored the transcriptional activity undergone by *T. virens* during two phases of symbiosis with maize; recognition of roots and after ingress into the root cortex.

**Results:**

We present a model of *T. virens* – maize interaction wherein *T. virens* experiences global repression of transcription upon recognition of maize roots and then induces expression of a broad spectrum of genes during colonization of maize roots. The genes expressed indicate that, during colonization of maize roots, *T. virens* modulates biosynthesis of phytohormone-like compounds, secretes a plant-environment specific array of cell wall degrading enzymes and secondary metabolites, remodels both actin-based and cell membrane structures, and shifts metabolic activity. We also highlight transcription factors and signal transduction genes important in future research seeking to unravel the molecular mechanisms of *T. virens* activity in maize roots.

**Conclusions:**

*T. virens* displays distinctly different transcriptional profiles between recognizing the presence of maize roots and active colonization of these roots. A though understanding of these processes will allow development of *T. virens* as a bio-control agent. Further, the publication of these datasets will target future research endeavors specifically to genes of interest when considering *T. virens* – maize symbiosis.

**Electronic supplementary material:**

The online version of this article (10.1186/s12864-019-5651-z) contains supplementary material, which is available to authorized users.

## Background

*Trichoderma* (teleomorph *Hypocrea*) is a well-established fungal genus that has been the subject of numerous reviews [1–4]. In brief, *Trichoderma* spp. are mycoparasitic, facultative plant-symbionts that colonize a broad range of plant root systems. *Trichoderma-*derived plant benefits include enhanced lateral root development, increased nutrient uptake, resistance to abiotic stressors such as heavy metals and reactive oxygen species, and priming plants for pathogen resistance via triggering of ISR (induced systemic resistance). Consequently, *Trichoderma* spp. are widely used as agricultural bio-control agents. While new molecular investigative techniques have exponentially advanced our knowledge of *Trichoderma*, much remains to be discovered on the mechanisms by which *Trichoderma* spp. affect plants.

It is understood that *Trichoderma* colonizes plant roots in stages: first, actively growing towards roots indicative of root recognition, then growing externally on the root surface, followed by ingress in to the root cortex [5, 6]. A recent study by Nogueira-Lopez et al. [7] suggested that *T. virens* may colonize intracellularly and locate within the periplasmic space of the plant cell. During *T. virens* attachment to and invasion of plant root cells hydrophobin and cell wall degrading enzymes play an important role [8]. There is no detailed model, however, for the which particular enzymes and extracellular structures are used in root colonization and how they are regulated.

Once inside the plant, *Trichoderma* spp. effect change on phytohormone levels, specifically of phytohormones jasmonic acid (JA) and salicylic acid (SA) which pay central roles in orchestrating plant defense. It has been shown that *T. virens* generally suppresses SA in early colonization and then enhances JA levels to mediate ISR [9–12]. Plant growth hormones, i.e. indole-3-acetic acid (IAA), also have a correlational response to *Trichoderma* colonization of plant roots [13]. The molecular mechanisms behind *T. virens* influence on these phytohormones are a subject of current interest.

Many secreted proteins, metabolites, and enzymes also are known to play roles in *Trichoderma* – plant symbiosis. SM1 is a secreted protein of *T. virens* which suppresses the maize gene *ZmLOX3* to stimulate ISR [14–16]. Peptaibols, produced from non-ribosomal protein synthases, can affect both plant growth and disease resistance [17, 18]. Cell wall degrading enzymes (CWDE) are utilized for colonization – these also have current biotechnology applications. For example, CWDE isolated from *T. reesei* are already used for potent cocktails to degrade biofuel feedstock [19]. Continued exploration of the secondary metabolites, secreted proteins, and enzymes which *Trichoderma* utilizes within a plant host is important to informing future bio-industrial products. For example, supporting crop growth on marginal land would be benefitted by fostering *Trichoderma’s* ability to mitigate abiotic stress on plants, and optimization of bio-control agents requires a thorough understanding of how influential proteins are regulated and secreted.

In this study we sought to accelerate research targeted at *Trichoderma* plant interactions by using RNA-sequencing to conduct a comprehensive survey of the transcriptional activity experienced by *T. virens* co-cultivated with maize (*Zea mays*) during the recognition and after the ingress stages of root colonization. We anticipate that this study will accomplish two purposes. One, it will highlight important processes undertaken by *T. virens* for a productive root-symbiont relationship. Two, it will detail specific enzymes, proteins, metabolites, and other molecules which *T. virens* utilizes in the maize host, thus increasing precision of future studies.

## Results and discussion

### Differential expression analysis

The *Trichoderma virens* transcriptome was analyzed after 6 and 30 h of co-cultivation with maize. These timepoints were defined, respectively, as the “Recognition” phase, wherein *T. virens* was visibly growing towards maize but had not yet penetrated the roots, and the “Colonization” phase, wherein *T. virens* had made ingress into the maize root (see Methods and Additional file 1: Figure S2). Differential expression (DE) analysis was used to analyze each transcriptome. *T. virens* gene expression during Recognition was compared to gene expression of *T. virens* grown for 6 h alone; likewise, *T. virens* gene expression during Colonization was compared to *T. virens* growth for 30 h alone. This was done to minimize the impact of fungal aging and circadian rhythm on the DE analysis. The terminology ‘repressed’ is used to describe genes with a negative log_2_fold change, i.e. were less abundant when *T. virens* was grown with maize, and ‘enhanced’ to describe genes with a positive log_2_fold change, i.e. were more abundant when *T. virens* was co-cultivated with maize. A full list of genes with log_2_fold changes and FPKM (fragments per kilobase of transcript per million reads) values can be found in Additional file 2: Data file S1. A differentially expressed gene (DEG) was considered significant if it had a log_2_fold change of ±1.5 over the control sample and a false-discovery adjusted *p*-value of < 0.05 (See Additional file 3: Data file S2 for list of all DEGs so discovered). The RNA-seq based DE analysis was validated with RT-qPCR (Additional file 4: Figure S1). Log_2_fold change calculated as calculated by RT-qPCR and the DE analysis were consistent in terms of direction (i.e. DEGs repressed in the DE analysis were also repressed when measured by RT-qPCR). The magnitude of the log_2_fold change was not as well conserved. This is explicable by the differences in cDNA preparation and that the tissue used for RT-qPCR had been stored for a year, abet at − 80 °C (See Methods). Pearson correlation, however, was 0.75 when plotting log_2_fold changes obtained by DE vs RT-qPCR.

### Differential expression overview

**Fig. 1:**
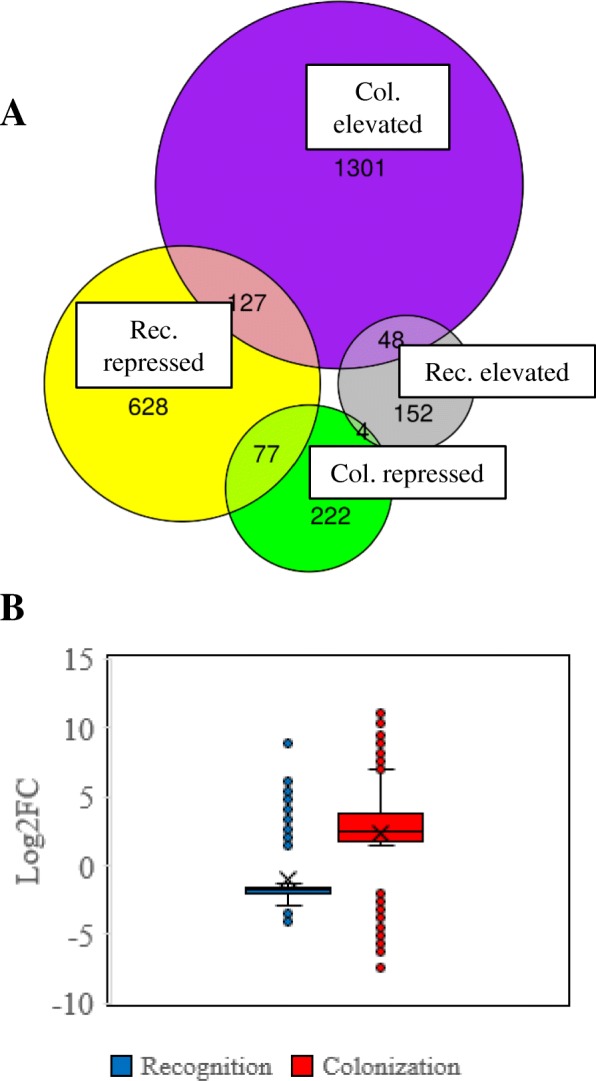
Recognition and Colonization datasets bear little similarity. **a** Venn diagram showing the number of DEGs which overlap between the Recognition and Colonization dataset. **b** Boxplot of the range of log_2_fold changes occurring in each dataset. Students t-test shows a significant difference between the range of log_2_fold changes in the datasets, < 0.00001 *p*-value

*T. virens* altered expression of 6% of its genome during the Recognition phase and 12% during the Colonization phase. The altered transcriptomes during each phase represent different subsets of the *T. virens* genome as there was little commonality between DEGs from the Recognition and Colonization datasets (Fig. 1a). Only 204 DEGs were constitutively repressed or enhanced from Recognition to Colonization. Further, the range of log_2_fold change was significantly different between the Recognition and Colonization phases, with the Recognition phase DEGs being generally repressed and Colonization DEGs being generally enhanced (Fig. 1b).

For downstream analysis each DEG was manually sorted into a functional group (See Methods and Additional file 3: Data file S2). A total of 347 and 679 genes for Recognition and Colonization, respectively, could not be placed into any category due to lack of information or poor characterization. Removal of No KOG/poor characterization DEGs left 450 significant DEGs in the Recognition dataset and 875 significant DEGs in the Colonization dataset. Even within individual functional categories, the trend of DEG repression during Recognition and elevation during Colonization was conserved. Below we use the expression of DEGs within each functional category to build a hypothetical framework of *T. virens* activity with maize. For ease of presentation we break down our analysis into four sections: Plant-Related Activity, Environmental Interaction, Fungal Internal Processes, and Fungal Metabolism and Energy.

#### Plant-related activity

##### Plant cell wall degrading enzyme DEGs

A total of 28 and 88 DEGs were annotated as being carbohydrate activated enzymes (CAZ) in Recognition and Colonization, respectively. These DEGs ranged across multiple glycosyl hydrolase/transferase families. The importance of glycosyl hydrolases in *T. virens* colonization of maize and tomato has been noted in previous studies [20, 21]. We focus here on the CAZ specifically annotated as being cell wall degrading enzymes (CWDE), including chitinases, cellulases, pectinases, arabinofuranosidases, laccases and other lignin-degrading enzymes [22]. While these CWDE’s may have been involved in mycoparatism or self-cell wall remodeling, the presence of lignin and pectin degrading enzymes suggests catabolism of plant cell walls was likely occurring [22, 23]. Whether this catabolism functioned to aid *T. virens* ingress of root intercellular spaces or served as a nutritional source *in planta* is unclear [22–24].

**Fig. 2:**
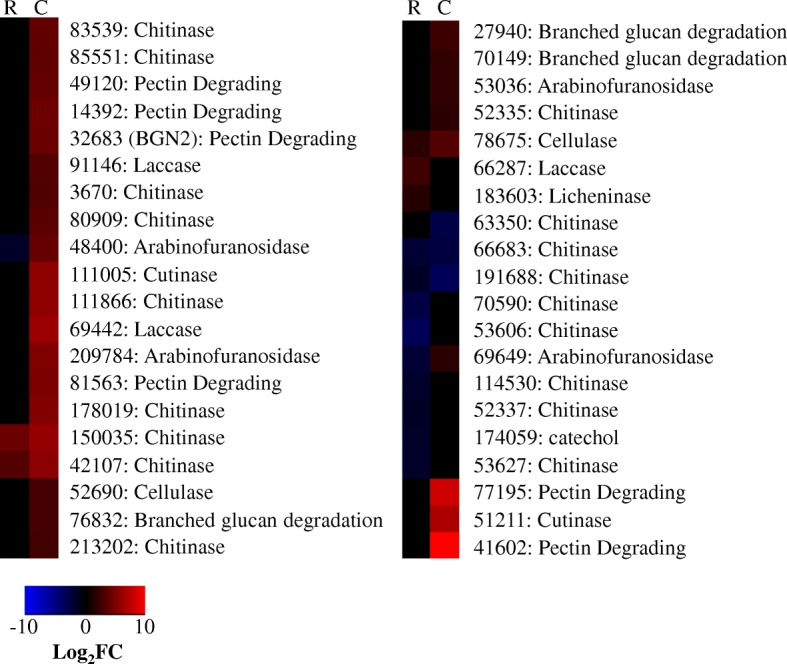
Heatmap of cell wall degrading enzymes. Heatmap compares the log_2_fold change for a given gene during Recognition (R) and Colonization (C). Red indicates more abundant gene expression during growth with maize and blue indicates less abundant gene expression. Black indicates a gene not considered significant in our analysis. Labels give the *T. virens* Gv29–8 gene number as annotated the JGI database version 2 and the predicted gene function

In general, the CWDE’s were either not significant or repressed during Recognition; coordinating well with the data that the fungus had not yet made ingress into the plant roots. The repression of specific CWDE may also be linked to avoidance of plant defenses. CWDE’s have been shown to elicit plant defense as PAMPs in other organisms [25–28]. Five putative CWDE’s were, however, still enhanced during Recognition. Two of these were chitinases also enhanced during Colonization (DEGs 150035 and 42107). A further two chitinases (DEGs 63350 and 66683) were repressed in both datasets (Fig. 2).

During *T. virens* colonization of maize, pectinases appeared to play a highly important role. The highest transcriptional enhancement was a pectin-degrading enzyme (DEG 41602), which expression was 11 log_2_fold more abundant than the control. Another pectinase (DEG 77195) was expressed 8 log_2_fold over the control. Five additional pectinase DEGs were enhanced during colonization, albeit to a lesser degree. Although *Trichoderma* genomes have comparatively few pectinases in relation to other CAZ and proteases, the *T. virens* genome contains pectinases capable of degrading multiple pectin architypes, indicative of the ability to colonize many different plant hosts [29–31]. Thus, the subset of pectinases enhanced in this study may represent *T. virens* response to maize on a host specific level. Morán-Diez et al. [20] also found that the pectinase 77195 was enhanced during colonization of maize roots, but not tomato roots.

Additional DEGs found significant during *T. virens* colonization of maize were: four arabinofuranosidases, two cutinases, two cellulases, two laccases, ten chitinases, and three enzymes which degrade complex, branched glucans. The number of CWDE expressed by *T. virens* may seem redundant [32]: however, research has shown that breakdown of plant cell walls is a synergistic process involving many degrading enzymes [22]. Indeed, Margolles-Clark et al. [33] presented evidence that plant cell wall substrate will provoke expression of cellulases and hemicelluloses to a differential extent, providing evidence that break-down of plant cell wall is a tightly regulated multi-component processes.

##### Phytohormone-like biosynthetic DEGs

The influence of *T. virens* on host phytohormones has been much studied. While the molecular mechanisms remain a mystery, multiple studies have correlational evidence that *T. virens* stimulates a shift in phytohormone production. A model is emerging where *Trichoderma* spp. need to limit salicylic acid (SA) production to colonize plant roots [10], and subsequently boost jasmonic acid (JA) [11]. Interestingly, other endophytic fungi also repress plant SA production and have strain-dependent effects on JA [9]. Fungal effectors seem to play a large role in the mediation of SA/JA as application of *T. virens* spores versus filtrate to a plant hosts results in differential induction of SA or JA [12, 34]. Most research on *T. virens* mediation of phytohormone levels measures either the hormone level *in planta* or hormone-dependent gene expression as a reflection of hormone level. This study uniquely shows that, during maize colonization, *T. virens* has transcripts for genes putatively biosynthesizing or modulating phytohormones.

**Fig. 3:**
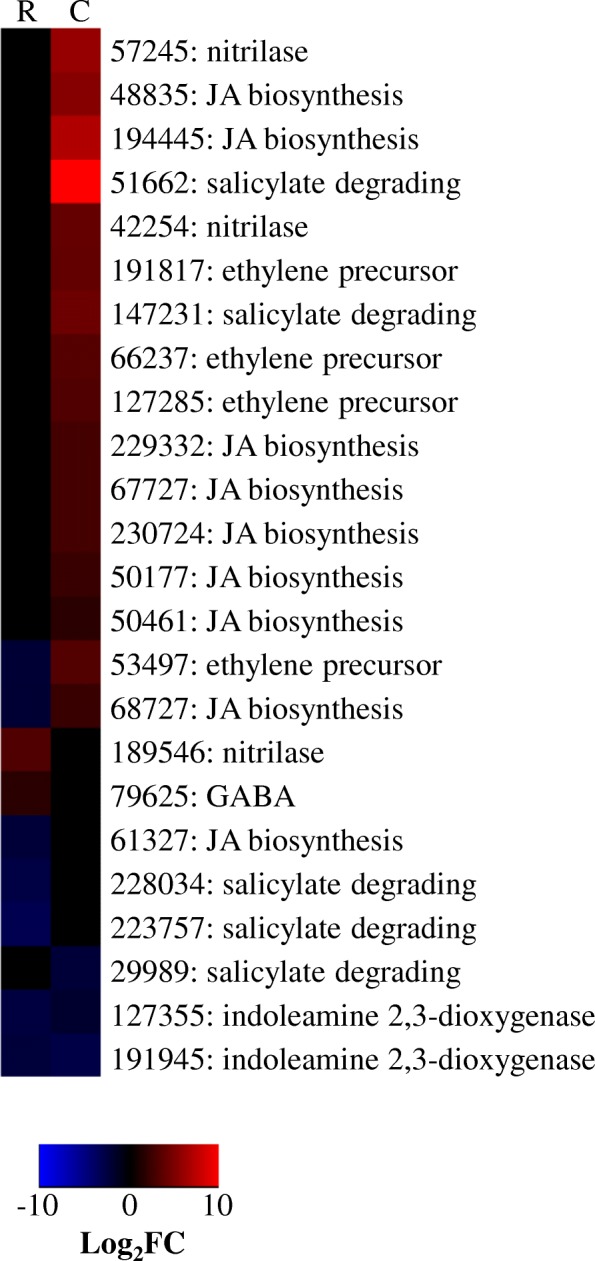
Heatmap showing alternative regulation of genes involved in plant hormone synthesis / degradation. Heatmap compares the log_2_fold change for a given gene during Recognition (R) and Colonization (C). Red indicates more abundant gene expression during growth with maize and blue indicates less abundant gene expression. Black indicates a gene not considered significant in our analysis. Labels give the *T. virens* Gv29–8 gene number as annotated the JGI database version 2 and the predicted gene function

At Recognition two salicylate degrading genes (DEGs 228,034 and 223,757) were repressed; whereas, during active colonization of maize, the transcription of two different salicylate degrading enzymes were enhanced (DEGs 51,662 and 147,231). 51,662 was particularly highly enhanced with an abundance at 10 log_2_fold more than the control. At Colonization 7 DEGs encoding JA biosynthesis enzymes were enhanced (Fig. 3). Of these genes, two (DEGs 68272 and 61327) were repressed at Recognition, indicative of repression of JA biosynthesis. We also note the transcription of a precursor to ethylene synthesis (DEG 66237) during Colonization. Further, there may be effects on indole-3-acetic acid (IAA), an auxin related to plant growth and development. Three nitrilases were enhanced DEGs which have the potential to produce an IAA precursor, indole-3-acetonitrile (Fig. 3).

##### Oxylipin biosynthetic DEGs

Association with maize prompted alternate expression of several oxylipins by *T. virens*. Note that many of the genes annotated as potentially being involved with oxylipin synthesis are unspecified monooxygenases or P450 CYP2 sub-family cytochromes. Their role in specific oxylipin synthesis will need to be verified by further research. Such research will be important to further unravel *Trichoderma*-plant interaction as oxylipins are prominent candidates for inter-kingdom signaling molecules [35] Lineolate acid epoxide derivatives have been shown to be involved in anti-fungal defense mechanisms, cross-talk between fungi and their plant hosts, and in JA biosynthesis [35–38]. Our analysis identifies 15 DEGs involved in lineolate acid metabolism. Arachidonate acid metabolism is another venue for oxylipin production, synthesizing HETE and DHET oxylipins. This study identifies 17 DEGs as potential producers of 20-HETE, 19(S)-HETE, 16(R)-HETE, 11,15,15-THETA, 11,12,15-THETA, 14,15-DHET, 11,12-DHET, 8,9-DHET, and/or 5,6-DHET (See Additional file 3: Data file S2; Oxylipins). DEG 190955 is of special interest as this monooxygenase has the potential to influence oxylipins production from either lineolate or arachidonate metabolism and was enhanced at both Recognition and Colonization, 3.6 and 8.7 log_2_fold, respectively.

##### Additional DEGs encoding putative plant-utilized compounds

Six DEGs involved in 4-coumarate-CoA synthesis were enhanced during *T. virens* colonization of maize roots (Additional file 3: Data file S2). 4-coumarate-CoA is the base molecule for synthesis of many plant secondary compounds, including flavonoids, isoflavonoids, and lignans [39]. Whether this corresponded to fungal biosynthesis and/or secretion of these molecules was outside the scope of this study, but an intriguing concept. Also seen were the elevation of catechol metabolic genes. Catechol is utilized by both plants and fungi. In plants catechol oxidase is often expressed when under stress from pathogenic attack [40]. Catechol dioxygenase has been shown to be a response of the fungal pathogen, *Cochliobolus heterostrophus*, to plant secreted phenolics [41]*.* The production of catechol may also be a by-product of salicylate degradation by fungi and bacteria [42, 43].

##### Nitrogen metabolism DEGs

**Fig. 4:**
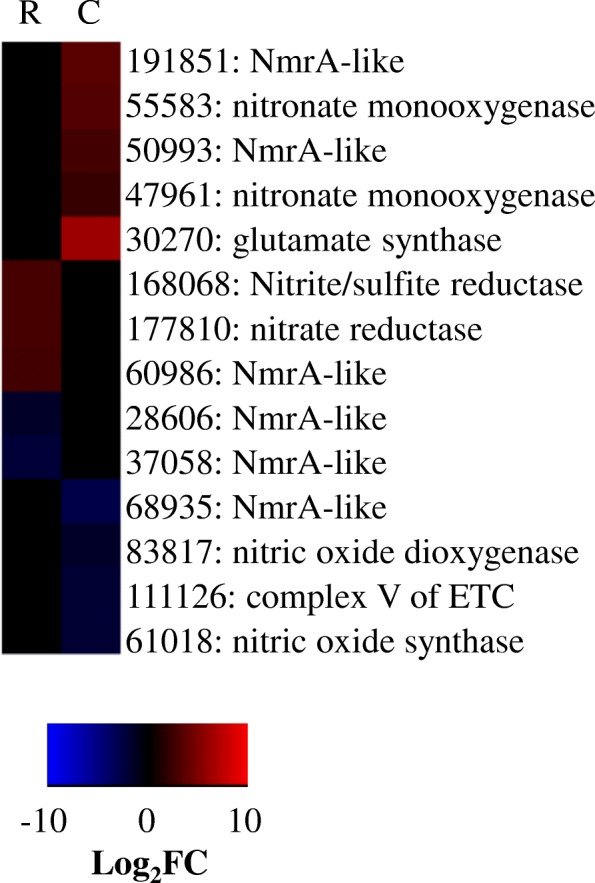
Heatmap of nitrogen activities. Heatmap compares the log_2_fold change for a given gene during Recognition (R) and Colonization (C). Red indicates more abundant gene expression during growth with maize and blue indicates less abundant gene expression. Black indicates a gene not considered significant in our analysis. Labels give the *T. virens* Gv29–8 gene number as annotated the JGI database version 2 and the predicted gene function

*Trichoderma* spp. can enhance plant nitrogen content [12], notably when co-inoculated onto plant hosts with nitrogen fixing bacteria [44]. In this study, *T. virens* appeared to alter nitrogen cycling activities starting with recognition of maize roots, during which time three NmrA-like transcripts were repressed, but two nitrite/nitrate reductases (DEGs 168068 and 177810) were enhanced. During Colonization two NmrA-like genes were enhanced (DEGs 50993 and 191851), while one was repressed (DEG 68935). Two nitronate monooxygenases were also enhanced (DEGs 47961 and 55583). Interestingly, there may have been some disfavor of nitric oxide at Colonization; both a nitric oxide dioxygenase (DEG 83817) and a nitric oxide synthase (DEG 61018) were repressed (Fig. 4). These genes could indicate repression of internal nitric oxide; however, Gupa et al. [45] demonstrated that *T. asperelloides* suppresses plant host nitric oxide levels in response to *Fusarium* attack. We speculate that *T. virens,* also has the potential to modify plant nitric oxide levels.

#### Environmental interactions

##### Defense-related DEGs

**Fig. 5:**
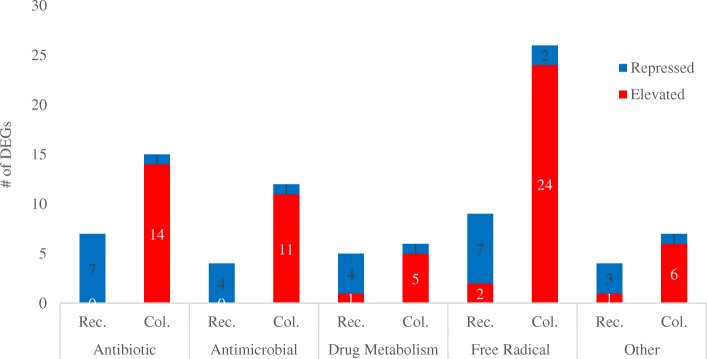
Defense DEGs are repressed during Recognition and enhanced during Colonization. Stacked bar graph shows the number of DEGs with defense-related functions which are repressed or enhanced during Recognition (Rec.) and Colonization (Col.). DEGs are sorted into potential activities, including Antibiotic (production or breakdown of antibiotics), Antimicrobial (production of compounds known to inhibit microbial growth, but are not known antibiotics), Drug Metabolism (DEGs involved in the production or transport of drugs), Free Radical (catalases, peroxidases, glutathione metabolic genes, i.e. those which may protect the fungus from free radical attack), and Other (putative defense DEGs, see Additional file 3: Data File S2)

The plant biome is a competitive environment, particularly the rhizosphere which hosts a plethora of competing microorganisms. This study, however, was conducted in a hydrophobic environment where competition was lacking. Despite this four putative Defense DEGs were enhanced during Recognition: catalase (88881), a glutathione-S-transferase (231671), an arsenic resistance protein (194498), and finally a DEG with a putative stress A/B barrel domain (34890). Both an iron permease (DEG 195287) and a ferric reductase were enhanced, and a gene predicted to remove iron from siderophores (DEG 147314), suggestive that *T. virens* initiation of iron-scavenging is stimulated by maize roots and not rhizosphere competition. Other metal transporters were affected as well; a Ni transport gene was enhanced, while a zinc transporter and a heavy metal transporter were repressed. While we did not expect to see evidence for increased antibiotic expression in the sterile hydroponic system, it was also unexpected that six beta-lactamases and five DEGs predicted to function in drug metabolism/transport were repressed in the presence of maize roots (Fig. 5). Conversely, at Colonization, antibiotic metabolism and production of anti-microbial compounds may be increased. This was evidenced in the elevation of DEGs involved in isoquinoline biosynthesis, beta-metallo-lactamases, fungalysins, and drug transport and resistance (also see Secondary Metabolite section). Additionally, DEGs were enhanced which indicate expression of conotoxin, colicin, and a cyanovirus-targeted toxin. *Trichoderma* spp. are well known to be resistant to many toxins [46], but the secretion of toxins *in planta* is less studied.

Reactive oxygen species (ROS) were likely prominent in the *in planta* environment as *T. virens* enhanced 16 glutathione-metabolism genes, 4 catalases, and 1 peroxidase. Note that 2 peroxidase genes were repressed (Fig. 5, Additional file 3: Data file S2). ROS are common in plant stress signaling (reviewed in ref. [47]), and plant defense systems [48–50]. ROS burst can also occur when a symbiotic microorganism colonizes a plant host, as noted by Santos et al. [51] with alfalfa and *Sinorhizobium meliloti*. *Trichoderma* spp. can help plants alleviate heavy-metal induced ROS accumulation by expression of glutathione enzymes [7, 52–54]. We hypothesize therefore that the expression of glutathione metabolic genes by *T. virens* in maize could serve a dual role of self-protection and plant health promotion.

##### Secondary metabolite production DEGs

**Fig. 6:**
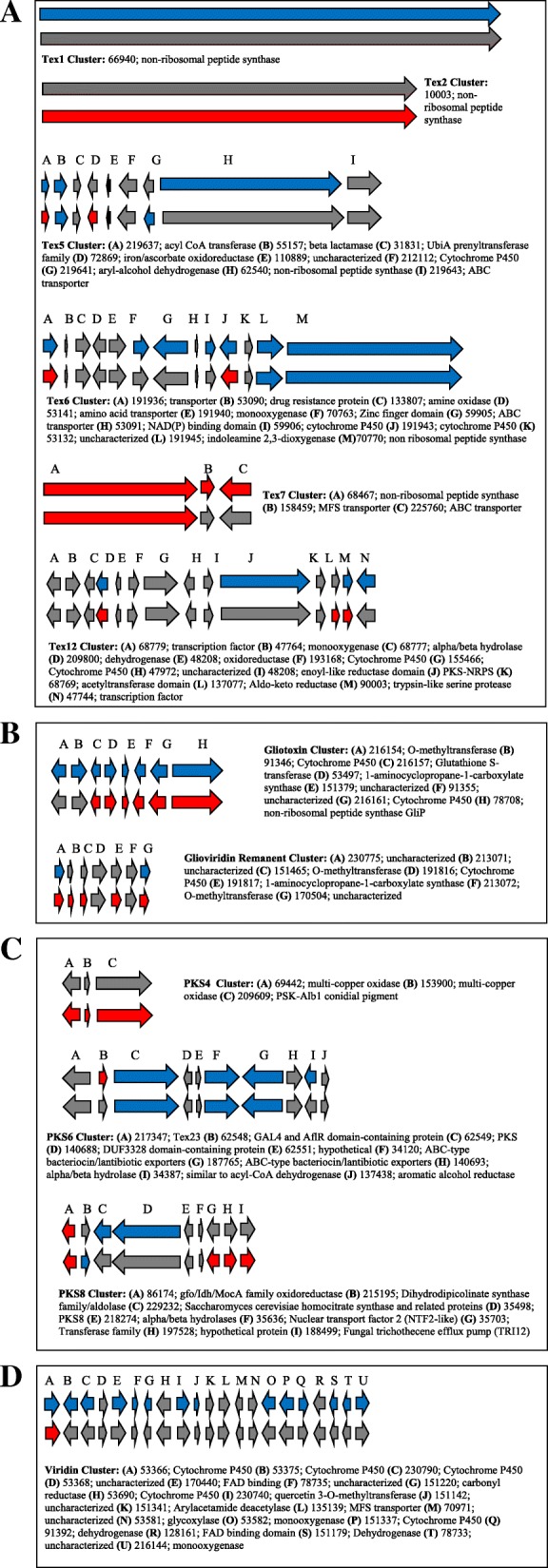
Gene Clusters involving (**a**) NRPS, (**b**) Gliotoxin, (**c**) PKS and (**d**) Viridin. Top row of arrows represents gene expression at Recognition, bottom row represents gene expression at Colonization. Red arrows indicated an enhanced log_2_fold change, blue a repressed log_2_fold change, and gray a non-significant log_2f_old change

*Trichoderma* spp. produce a plethora of secondary metabolites, both of high (peptaibols) and low (ETPs, terpenes, steroids and polyketides) molecular weight, and their genomes are rich in genes related to secondary metabolites biosynthesis and transport [1]. These secondary metabolites have widely-acknowledged roles in influencing plant growth, development, and disease resistance [2, 18, 55–57]. To conduct a comprehensive analysis of regulation of secondary metabolism-related genes and gene clusters during interactions of *Trichoderma* with roots, we created a database by identifying nearly all the genes putatively involved in secondary metabolism biosynthesis in *T. virens* (Additional file 5: Data file S3). This database of genes is unique in our analysis in that more genes were affected during Recognition than Colonization, although the trend of the majority of DEGs being repressed during Recognition was conserved. Note, since we wanted to capture any change in a gene cluster, we considered a gene significant if the p-adjust value was < 0.05, regardless of whether the log_2_fold change was ±1.5. Below we discuss which specific non-ribosomal peptide synthases (NRPS), polyketide synthases (PKS), and other secondary metabolites gene clusters were found significant in this analysis (Fig. 6).

The *tex1* gene cluster, synthesizing an 18-residue peptaibol, was repressed during the Recognition phase. This was unexpected; Viterbo et al. [17] demonstrated that *tex1* was expressed in association with cucumber seedlings and is involved in triggering ISR. During Colonization *tex1* reverted to basal level expression. Induction of the *tex5* cluster during Colonization was inferred by the very strong expression (5 log_2_fold) of two genes in this cluster: an aldo-keto reductase (DEG 219641) and an isopenicillin N synthase family oxygenase (DEG 72869). While two of the *tex5* cluster genes were repressed during Recognition this was only a mild effect, thus we hypothesize that *tex5* mainly functions during the Colonization phase. The *tex6* gene cluster was mostly repressed during Recognition. Both *tex5* and *tex6* NRP’s have unknown products; this analysis is the first to suggest a role for them in fungal-plant interactions. For *tex6* the repression during Recognition suggests a role in antagonistic activity. One gene in the *tex7* cluster was enhanced during Recognition, but all three genes were enhanced during Colonization. *Tex7* putatively codes for a 5-residue NRP. The *tex12* cluster is a NRSP-PKS hybrid. Like the *tex5/6* clusters, the product is unknown (Fig. 6a).

The *tex18* cluster is more commonly known as the gliotoxin biosynthesis cluster. Gliotoxin is an ETP compound copiously secreted by *T. virens* [58]. Typically, gliotoxin is strongly expressed for 16 h in fresh culture and can be detected in culture media for up to 48 h [58]. At Recognition (6 h of co-cultivation with maize) the cluster was very strongly repressed, with every gene in the cluster being less abundant in the presence of maize. At Colonization (30 h of co-cultivation) all but two genes in the cluster were significantly enhanced (Fig. 6b). *Trichoderma* “Q” strains are characterized by their production of gliotoxin while *Trichoderma* “P” strains produce another ETP, gliovirin. Our analysis detected strong elevation of expression of a remnant of the gliovirin cluster during Colonization. This gliovirin remnant cluster has 7 of the putative gliovirin biosynthesis genes, but lacked the main non-ribosomal peptide synthesis gene (Fig. 6b).

Among the PKS’s, cluster 6 was strongly repressed during Recognition. During Colonization the PKS 4 cluster was enhanced. This cluster biosynthesizes conidial pigments in *T. virens*. This was intriguing as under these growth conditions, *T. virens* should not have conidated. However, this gene has also been found to be responsible for general regulation of other PKSs and stress tolerance in *T. reesei* [59]. Indeed, PKS cluster 8 was also enhanced during Colonization; it is possible that the elevation of PKS4 functioned to regulated PKS 8 expression (Fig. 6c).

Recently a cluster of 21 genes, responsible for synthesizing viridins, was uncovered [60]. Viridin is rapidly converted to viridiol, a phytotoxic agent [60]. Of the 21 gene cluster, 14 were significantly repressed during Recognition (Fig. 6d). During Colonization only two viridin genes were enhanced; none were repressed. We speculate that *T. virens* production of viridin may be perceived by the maize roots as a damaging agent.

##### Small secreted cysteine-rich proteins (SSCP) DEGs

**Fig. 7:**
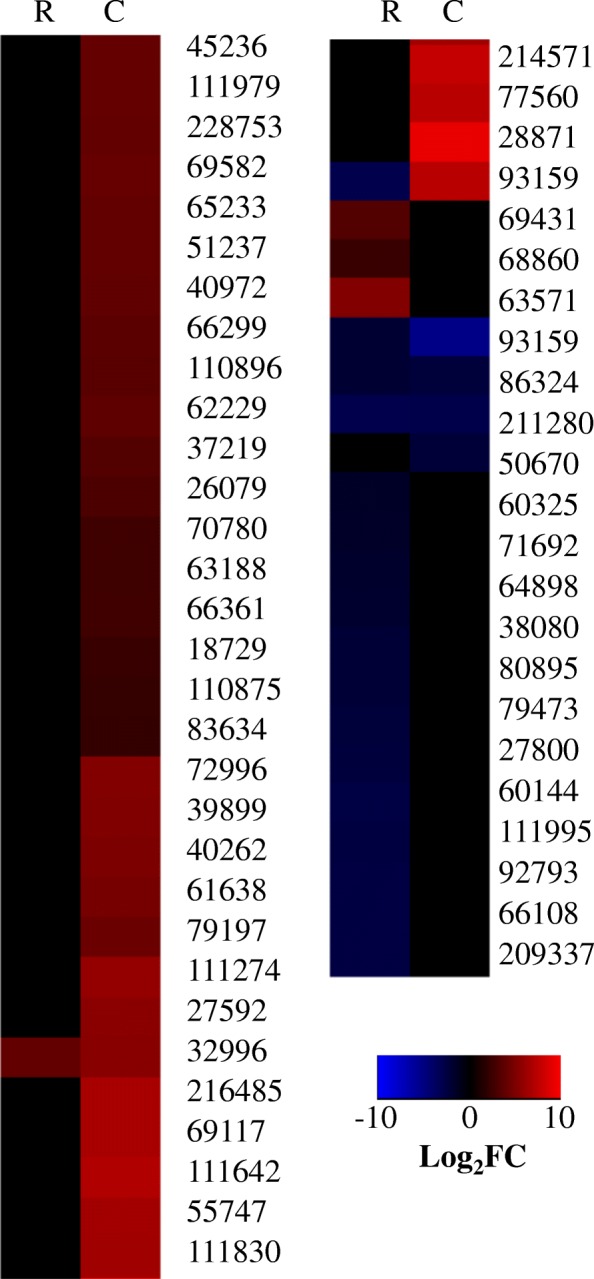
Heatmap compares the log_2_fold change for a given gene during Recognition (R) and Colonization (C). Red indicates more abundant gene expression during growth with maize and blue indicates less abundant gene expression. Black indicates a gene not considered significant in our analysis. Labels give the *T. virens* Gv29–8 gene number as annotated the JGI database version 2; all genes are putative SSCP’s

*T. virens* secretes many proteins, termed effectors, which function in *T. virens* bio-control abilities [16, 21]. Our analysis returned 55 SSCP (small secreted cysteine rich protein) DEGs, which were expressed considerably differently between the Recognition and Colonization phases (Fig. 7). Among these SSCP’s were two genes identified in previous research: MRSP1 (DEG 45236) and SM2 (DEG 111830); both were enhanced during Colonization. MRSP1 is a SSCP negatively regulated by a MAPK protein [61], TmkA. *TmkA* mutants are impacted in some mycoparatism activities [62]. MRSP1 may thus have some impact on *T. virens* direct bio-control properties. SM2 is a paralog of the well-studied SM1, a known trigger of plant induced system resistance (ISR) [16, 63]. SM2 may have a different function in stimulating plant defense than SM1. Research has shown that *T. virens sm2*, not SM1, is involved in induced defense against the maize pathogen *Cochliobolus heterostrophus*. It appears that SM1 activates SA-mediated response against biotrophs and hemi-biotrophs while SM2 triggers JA-mediated defense against necrotrophs [64]. As SSCPs may be potent effectors of plant defense [65] those SSCP DEGs constitutively affected during *T. virens* – maize co-cultivation were of interest. Three of these were continually repressed (DEGs 86324, 92434, and 211280). DEG 86324 is annotated as pH responsive and potentially affecting transcription factor activity. DEGs 92434 and 211280 are uncharacterized beyond putative SSCPs. One DEG (32996, having a lysozyme-like superfamily domain) was continually enhanced. DEG 93159 is annotated only as a putative SSCP and was oppositely regulated, being repressed − 2.0 log_2_fold at Recognition and enhanced 7.2 log_2_fold during Colonization.

##### Organismal morphology and mating DEGs

Shift from growth in liquid nutritive medium to *in planta* growth likely stimulates morphological change in *T. virens*. Upon *T. virens* recognition of maize roots this change is neither physically nor genetically evident; hyphae growing towards maize roots retained their filamentous appearance. During the Colonization phase, however, four genes predicted to have a role in actin-based cell structures were enhanced, as well as two actin cross-linking DEGs. It has been shown before that *T. harzianum* switches to a yeast-like morphology inside *Arabidopsis* roots [9] thus *T. virens* may do the same.

*T. virens* recombinant strain potential was affected by either co-cultivation with maize or exposure to the seed-borne endophytic maize microbiome, evidenced by the numerous heterokaryon incompatibility DEGs. At Recognition *T. virens* repressed 11 heterokaryon incompatibility proteins. Further, two genes annotated as both an oligopeptide transporters and sexual differentiation proteins, 189,327 and 84,303, were DEGs. During *T. virens* Colonization of maize 13 different heterokaryon incompatibility genes were enhanced, while two were repressed (Additional file 3: Data file S2).

##### Extracellular structures and Cell Wall remodeling DEGs

*T. virens* extracellular structures appeared to undergo swift remodeling starting upon exposure to maize roots and continuing through colonization. 31 extracellular / cell wall DEGs were found during Recognition and 53 during Colonization (Additional file 3: Data file S2). These genes represent hydrophobins, glycoprotein-associated, cell wall structures, adhesions, and glycolipid/glycophospholipid metabolic genes. Fungal cell wall and extracellular structures often mediate the initial fungal-microbe interaction [26–28, 38, 66]. The DEGs identified here will require additional empirical analysis to determine their roles in plant attachment and/or suppression/evasion of plant defenses.

**Fig. 8:**
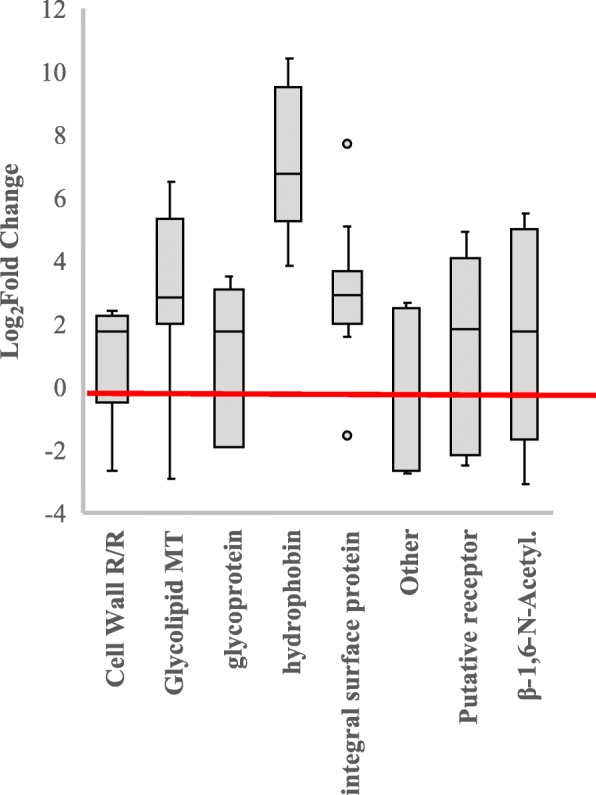
Hydrophobins were highly enhanced during Colonization. Box plot of the range of log_2_fold change for extracellular and cell wall structures during Colonization. Key; R/R = Replication and Repair; MT = Metabolism and Transport; β-1,6-N-Acetyl = β-1,6-N-Acetyltransferase

Hydrophobin DEGs were highly impacted within this functional category. Three hydrophobin-like genes were repressed at Recognition; two were ceratoulimins (DEGS 83985 and 9842), and the other (DEG 45185) was a lipocalin predicted to bind hydrophobins. One predicted hydrophobin was enhanced during Recognition, as was a putative extracellular protein (DEG 68031). During Colonization seven different hydrophobin encoding transcripts were enhanced. Although the highest number of extracellular DEGs were glycoproteins, DEGs annotation as being hydrophobins or hydrophobin-like had the greatest log_2_fold change in the differential expression analysis (Fig. 8). *TvHFB9a* (DEG 121648) and DEG 49849 were extremely enhanced; 10 and 9 log_2_fold, respectively. *TvHFB9a* has been tied to research demonstrating that hydrophobins can contribute to a protein biofilm layer on the fungal surface [67]. This protein layer was suggested by Bonazza et al. [67] to maintain moisture; however, hydrophobins have been shown to be critical in plant colonization and during abiotic stress for many fungi [8, 68, 69]. The failure of fungal hydrophobin mutants to colonize plant roots may be a structural issue – hydrophobins affect aerial hyphal growth as well as absorption to surfaces [28, 67, 70]. Askolin et al. [70] reported that two *T. reesei* hydrophobins, *hfb1* and *hfb2*, could not complement each other. Thus the 15 hydrophobin genes identified in our analysis cannot be assumed to have had redundant functions.

Of special interest were nine DEGs annotated as beta-1,6-N-acetylglucosaminyltransferases. These molecules directly modify the glycans present on the cell wall, which can serve as MAMP/PAMPS (Microbial or Pathogen Associated Molecular Patterns) recognized by the plant host [71]. Three of these transcripts were repressed at Recognition, possibly indicating evasion of host defense. DEG 216297 was continually repressed into Colonization as well. Further, two DEGs with LysM domains were repressed (DEG 222410) and enhanced (DEG 120125) during Colonization. These DEGS may have aided in fungal colonization by masking chitins which would otherwise act as plant defense elicitors [72, 73].

##### Cell membrane remodeling DEGs

**Fig. 9:**
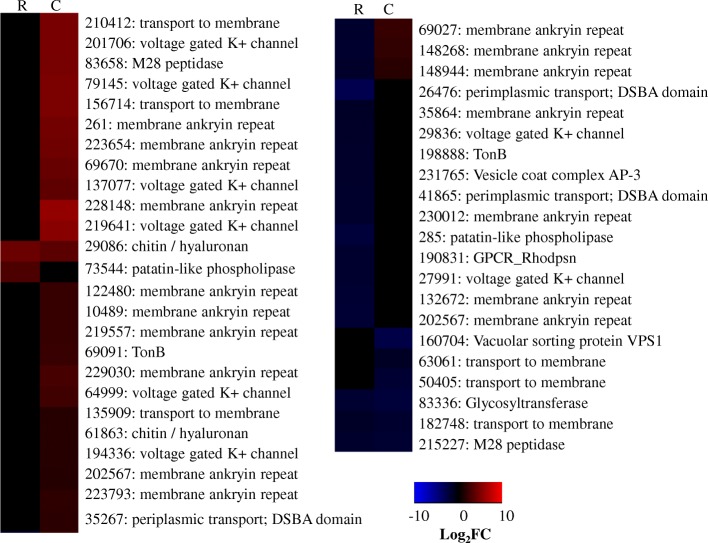
Heatmap compares the log_2_fold change for a given gene during Recognition (R) and Colonization (C). Red indicates more abundant gene expression during growth with maize and blue indicates less abundant gene expression. Black indicates a gene not considered significant in our analysis. Labels give the *T. virens* Gv29–8 gene number as annotated the JGI database version 2 and the predicted gene function

There was extensive evidence for the remodeling of cell membrane at *T. virens* recognition of maize roots. At Recognition 28 DEGs predicted to influence cell membrane were identified (Fig. 9). 20 of these DEGs were repressed. At Colonization 49 such DEGs were identified with 42 of these being enhanced (Fig. 9). DEGs predicted to influence cell membrane structures encoded membrane ankyrin repeat domains, phospholipases, permeases, integral membrane proteins, and channels / membrane anchoring or transport proteins. Of interest were two DEGs, repressed at Recognition, which are predicted to function in periplasmic transport and have DSBA domains. These domains can play a role in virulence, toxins, motility, and/or adhesion in bacterial pathogens [74]. We hypothesize repression of these genes resulted in outer membrane composition remodeling to evade triggering of host defenses prior to colonization. During the colonization phase we noted that six voltage gated K+ channel transcripts were enhanced. These may not have influenced membrane structure but do indicate remolding of membrane permeability (Fig. 9).

#### Impact on fungal internal processes

*T. virens* extracellular changes must be driven by internal genomic processes. For DEGs functioning in genomic functional groups such as Translation and DNA Repair/Replication, little change was noted. Several DEGs were altered which function in Transport and Vesicle formation, but these genes will have to be empirically studied to understand their function as well as the ten heat shock proteins repressed during Recognition (Additional file 3: Data file S2). Vast alterations were seen, however, in Transcription factors, Signal Transduction, and Post-Transcriptional/Translational Modification. These are described below.

##### Transcription factor DEGs

95 putative transcription factors were significant in our analysis between both fungal phases. The regulon of many of these transcription factors is unknown. Three DEGs were noted which belong to the Myb superfamily (DEGs 67860, 9220, and 130839). This superfamily of transcription factors is often active in plant development [75]. Four transcription factors had DEAD-box motifs, indicating potential involvement in RNA metabolism [76] (Additional file 3: Data file S2).

##### Signal transduction mechanism DEGs

**Fig. 10:**
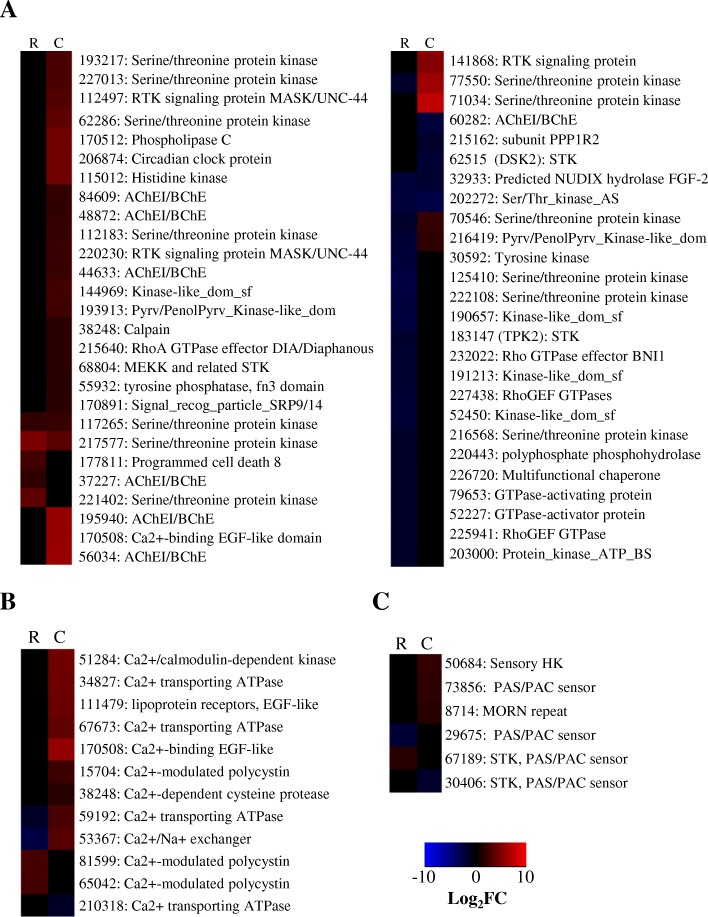
Signaling DEGs are important in *T. virens* maize symbiosis. Heatmaps compare the log_2_fold change for a given gene during Recognition (R) and Colonization (C). **a** shows putative signaling genes, **b** shows calcium dependent DEGs and **c** shows signal DEGs linked to environmental sensing. Red indicates more abundant gene expression during growth with maize and blue indicates less abundant gene expression. Black indicates a gene not considered significant in our analysis. Labels give the *T. virens* Gv29–8 gene number as annotated the JGI database version 2 and the predicted gene function

*T. virens* underwent re-programming of the signal transduction transcriptome within 6 h of co-cultivation with maize roots (Fig. 10). At the Recognition phase 35 DEGs were identified which had putative roles in signal transduction. Nine serine/threonine kinases (STK) were identified; four of which were also DEGs during Colonization (DEGs 117,265, 217,577, 70,546, and 77,550). DEG 29675 is a putative signal transduction gene of interest as this gene codes for a PAS/PAC domain, involved in environmental sensing, and a putative DNA binding domain.

An additional six STKs were identified as DEGs specifically during Colonization of maize roots; five of these were enhanced while only one was repressed. STKs in *Trichoderma* are best studied in the context of MAPKs (mitogen activated protein kinases). Roles for MAPKs have been found in hyphal growth, conidiation, mycoparatism, and mediation of ISR [4, 77–79]. Each MAPK signaling cascade appears to be specific to a particular process thus the STKs in our study could represent uniquely plant associated signal transduction mechanisms.

Another three DEGs with phosphatase domains and three receptor tyrosine kinases (RTK) with ankyrin repeat domains were also up-regulated during Colonization. Three additional signal transducing genes which bear further scrutiny were enhanced at *T. virens* colonization; DEG 50684 is annotated as a histidine kinase response regulator, DEG 227013 has a helix-turn-helix domain suggestive of a DNA-binding response regulator, and DEG 73856 has an ENVOY domain predicted to influence genes involved in light tolerance and cellulase. Concerning genes which could be involved in environmental perception leading to internal signal replay during *T. virens* colonization, we found DEG 8714 is both predicted to be a signal transducing gene and is predicted to link proteins to the outer membrane. A signal transduction DEG with a PAS domain (30406) was one of the few repressed signal transducing genes repressed at Colonization (Fig. 10).

This study highlights calcium as an important secondary messenger during *T. virens* – maize symbiosis (Fig. 10). Calcium was previously shown to be an important signaling molecule between *T. atroviridie* and plant cells [80]. Plant cells are also known to use calcium as intercellular messengers [81]. In our study, ten signal transduction genes annotated as calcium modulated or dependent were identified as DEGs. Three of these DEGs were enhanced during Recognition; one was repressed. The remaining six DEGs were up-regulated during *T. virens* – maize Colonization, four of which belong to the EF-hand superfamily indicative of direct calcium binding. Further, three calcium transporting genes were enhanced DEGs during Colonization. Of these DEG 59192 was significantly repressed during Recognition. Three calcium dependent genes were significant in both Recognition and Colonization and thus potentially play a role in signaling for the lifestyle switch required for *T. virens* to shift from growth in nutritive liquid media to growth in maize roots: DEG 13560(a C-type lectin), DEG 53367 (a Ca2+/Na exchangers), and DEG 59192 (calcium transporting).

##### Post-transcriptional/translational DEGs during colonization

Not all genetic changes can be captured in an RNA-Seq analysis as transcriptional regulation does not always reflect translated proteome. Therefore, it is important to pay attention to post-transcriptional/translation processes. Two ribonucleases were highlighted in our analysis which may function in post-transcriptional modification (DEG 47489 was enhanced at Recognition and Colonization; DEG 60427 was enhanced at Colonization). During Colonization, four co-splicing activators, two genes associated with RNA silencing, a dsRNA Dicer gene, and a T2 ribonuclease were enhanced. Oddly, a single gene functioning in adding the 5′ mRNA cap was repressed at both Recognition and Colonization (Additional file 3: Data file S2).

Post-translational modification occurred primarily during the Colonization phase. Only four genes which may modify amino acid residues were DEGs at Recognition. At Colonization eleven such genes were enhanced. A further four peptidases which may cleave polypeptides and protein precursors were enhanced at Colonization as well. General protein turn-over also appeared to be increased during *T. virens-*maize colonization; four peptidases with polypeptide protein precursor cleavage activity and two genes with protein turn-over activity were enhanced. DEG 2166625 is a ubiquitin hydrolase, cleaving polypeptide bonds with less than 60 amino acid residue and was repressed at Recognition and enhanced at Colonization. This gene may function in post-translational control of small secreted polypeptides. Finally, we note the differential activity of four genes with IBR and half RING finger domains, suggestive of modulation of both protein turnover and transcriptional activity. All four were enhanced during Colonization; one was repressed at Recognition (Additional file 3: Data File S2).

#### Fungal metabolism and energy

**Fig. 11:**
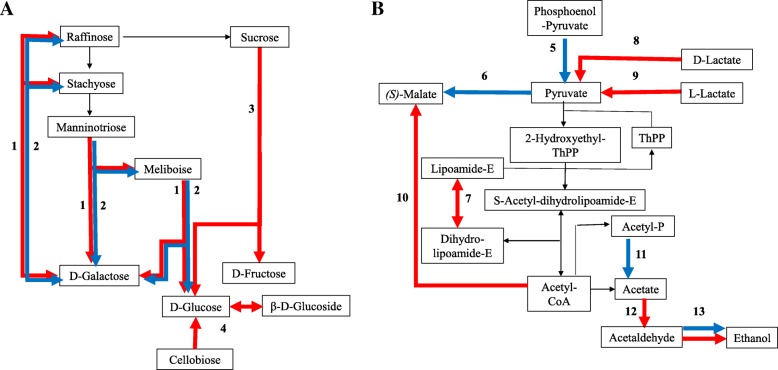
Alterations in pyruvate and sucrose metabolism during Colonization. Metabolic maps show putative pathways in sucrose (**a**) and pyruvate (**b**) metabolism in *Trichoderma* spp. Red arrows indicate a process enhanced during the Colonization phase and blue arrows indicate repressed processes. Each process is numerically labeled. The table indicates which individual DEGs support each numbered process, their log_2_fold change, KEGG Enzyme Class, and annotation

Plant-hosts provide a rich array of metabolites for fungal consumption. Consequently, many DEGs were classified as having metabolic function (Additional file 3: Data File S2). Analysis of these DEGs suggests that *T. virens* was converting plant produced sucrose to fructose or glucose and then to pyruvate (Fig. 11 and Table 1). Transcription of sucrose metabolic genes during *T. virens* – maize colonization was expected as sucrose is a key ingredient in *T. virens* – plant interactions [82–84]. Sucrose metabolic genes, however, were not altered until after root recognition. This would suggest that sucrose is not perceived by the fungus as an indicator of plant roots. Also unexpected was the abundance of transcripts capable of converting pyruvate into ethanol during colonization (Fig. 11). Such metabolite activity should be explored for potential in bio-fuel production.

**Table 1:** Details for gene involved in the processes highlighted in Fig. 11

Area	Gene #	Log_2_FC	KEGG EC	Additional Annotation
1	41607	3.01	3.2.1.22	Melibiase
1	27922	2.46	–	Melibiase
1	76590	6.63	–	Melibiase
2	39936	−1.96	–	Melibiase
3	45446	2.61	3.2.1.20	Maltase glucoamylase, GH family 31
3	53515	1.92	3.2.1.20	Alpha-amylase, GH family 13
3	48285	1.74	3.2.1.20	Alpha glucosidase, GH family 31
3	34797	5.16	3.2.1.20	Alpha glucosidase, GH family 31
4	83777	4.73	3.2.1.21	beta-glucosidase
4	29366	5.83	3.2.1.21	beta-glucosidase
4	41347	3.77	3.2.1.21	beta-glucosidase
4	192759	2.64	3.2.1.21	beta-glucosidase
4	151663	2.79	3.2.1.21	beta-glucosidase
4	188402	3.39	3.2.1.21	beta-glucosidase
4	58670	4.48	3.2.1.21	beta-glucosidase
4	200358	6.03	3.2.1.21	beta-glucosidase
5	51808	−1.63	2.7.9.2	Pyruvate, water dikninase
6	140682	−1.83	1.1.1.40	NADP+-dependent malic enzyme
7	170560	2.96	1.8.1.4	Dihydrolipoamide dehydrogenase
8	43109	1.67	1.1.2.4	D-lactate dehydrogenase)
9	32483	2.90	1.1.2.3	L-lactate dehydrogenase
9	88637	2.56	1.1.2.3	L-lactate dehydrogenase
10	89424	1.97	2.3.3.9	Malate synthase
11	75459	−2.05	2.7.2.1	Acetate kinase
12	67893	5.35	1.2.1.3	Aldehyde dehydrogenase
12	56585	4.07	1.2.1.3	Aldehyde dehydrogenase
12	74949	1.94	1.2.1.3	Aldehyde dehydrogenase
12	82254	2.22*	1.2.1.3	Aldehyde dehydrongase
13	88512	5.07	1.1.1.2	Alcohol dehydrogenase
13	28993	3.30	1.1.1.2	Alcohol dehydrogenase
13	34930	3.26	1.1.1.2	Alcohol dehydrogenase
13	78522	2.51	1.1.1.2	Alcohol dehydrogenase
13	217031	2.12	1.1.1.2	Alcohol dehydrogenase
13	82626	2.05	1.1.1.2	Alcohol dehydrogenase
13	215323	−1.82	1.1.1.2	Alcohol dehydrogenase

The alterations to lipid metabolism are not as clear cut. Sphingophospholipid metabolism seems to have been enhanced during Colonization, when three ceramide synthesis DEGs were enhanced. Sphingolipids function in plant defensive systems and are well-known in plant pathogen interactions (reviewed in ref. [85]). The fate of hexadecanoyl-CoA may diverge between *T. virens* Recognition and Colonization of maize roots. Transcripts encoding hexadecanoyl-CoA synthesis were enhanced during both phases, but genes encoding enzymes for the breakdown of hexadecanoyl-CoA were repressed during Recognition (Additional file 3: Data file S2). We hypothesize that hexadecanoyl-CoA was diverted to sphingophospholipid metabolism during Recognition, supporting the initiation of membrane remodeling [86].

Sulfur metabolic processes were important during the colonization phase. This was inferred based on six DEGs in taurine metabolism, which may be catabolized under sulfur starvation conditions [87]. We also noted a highly enhanced sulfate uptake DEG (193580, 5 log_2_fold increase), and two sulfite oxidases that were very highly enhanced, 6 and 8 log_2_fold for DEGs 215055 and 231246, respectively. The need for sulfur during Colonization may simply be a consequence of protein formation, as *T. virens* alters its’ secondary metabolite production within maize roots (see Secondary Metabolite section). This may also, however, be related to expression of the cellulases [88] that *T. virens* also utilizes during Colonization (see Plant Cell Wall Degrading Enzyme DEGs section).

Elevation of five dihydrodipicolinates (rate limiting step of lysine biosynthesis) and seven serine biosynthetic DEGs indicates a need for these two amino acids during *T. virens* – maize colonization. The cause for this likely lies in the translation of specific proteins, but more analysis is required.

Finally, we report that oxidative phosphorylation may have been depressed in the hydroponic maize rhizosphere which *T. virens* encountered during the Recognition phase. Eleven NADH oxidoreductases were repressed. Five of these are annotated as zinc binding NADH oxidoreductases and six are specifically involved in Complex I of oxidative phosphorylation. Once *T. virens* had colonized the plants five of these NADH oxidoreductases became enhanced again, along with an addition seven different NADH oxidoreductase transcripts. During Colonization there was also the elevation of several phenol monooxygenases, predicted to be involved in ubiquinone synthesis (Additional file 3: Data File S2).

## Conclusion

We present here the most comprehensive genomic analysis, to date, of the *T. virens* transcriptome during the recognition and subsequent colonization of maize roots. We present data which indicates that when *T. virens* first ‘recognizes’ the presence of maize roots the fungus represses transcription of genes across a broad spectrum of activity. We hypothesize that this is done either to avoid stimulation of plant defenses or to conserve energy required for the enhanced transcription of proteins required for root colonization. During actual colonization of maize roots, *T. virens* enhances expression of genes with a wide array of functions, including but not limited to: plant cell wall degradation, reactive oxygen species, phytohormone biosynthesis, secondary metabolites, metabolism, and signal transduction. These genes are likely necessary to facilitate the entry of *T. virens* into the maize root and persist in that altered environment. The genetic evidence presented for direct transcription of phytohormones, jasmonic acid in particular, may provide new scaffolding for unraveling the mechanisms by which *T. virens* affects plant hormone signaling. Further, we show that *T. virens* likely undergoes morphological changes within the root cortex in terms of both actin-based structure and cell wall/membrane remolding. The reason for this change is yet unknown but may reflect spatial constraints existing within the plant environment.

We anticipate that the full value of this dataset will be revealed over time as the genes identified here will provide a basis for increasingly targeted studies, in terms of microarrays, RT-qPCR, and genetic manipulation aimed at better understanding of the agronomically important symbiosis between *T. virens* and maize. Further, comparison of transcriptomic studies will push forward our understanding of plant-microbe interactions. For example, Morán-Diez et al. [20] conducted a microarray analysis comparing the transcriptomic responses of *T. virens* colonizing tomato roots versus maize roots after 72 h. This microarray presented data that *T. virens* does, in fact, respond to different plant hosts in a transcriptionally distinct manner. Comparison with the Colonization DEGs (30 h) in our dataset show 30% similarity with the genes Morán-Diez et al. [20] discovered as enhanced in maize (cultivar Silver Queen). Yet we also have a 30% similarity with those enhanced in tomato (cultivar Moneymaker). A recent study by Nogueria-Lopez et al. [7] identified 43 proteins secreted by *T. virens* into maize apoplast (cultivar 34H31) after 5 days. Only five of these proteins are transcriptionally enhanced during our Colonization dataset (DEGs 215514, 74949, 29366, 71600, and 53497). All three studies (this one, 7, and 20), however, indicate that mitigation of reactive oxygen species, abiotic stress, and production of glycosyl hydrolases are important in *T. virens*– maize interaction, even though the exact gene/protein utilized is different.

Several ideas can be extrapolated from this brief comparison. One, *T. virens* responds to plant hosts in a strain dependent manner. Two, *T. virens* – maize interaction is quite dynamic over time in terms of transcriptional activity and secreted proteins. Three, the overall mechanisms of plant interactions are conserved between plant hosts and individual genes have adapted to specific hosts. To validate these hypothesizes rigorous meta-analysis is needed of ‘omics data on *T. virens* – maize interactions. This requires more published data in terms of *T. virens*-maize association, on a strain level and with respect to hours/days of colonization. Such information will greatly enhance bio-engineering of bio-control strains to specific hosts and development of *Trichoderma* based enzymes for specific purposes.

## Methods

### Hydroponic set-up

B73 wild-type maize seeds used in this study were locally grown and provided by Dr. Kolomiets.

The wild-type strain (Gv29–8) of *T. virens* was isolated from a sandy loam soil cultivated with cotton plants in Texas and is deposited at the Fungal Genetics Stock Center, with genome sequence and annotation available at the JGI portal, version 2 (https://genome.jgi.doe.gov/TriviGv29_8_2/TriviGv29_8_2.home.html). Prior to placement of maize seedlings in the hydroponic system, the seeds were sterilized with a 70% ethanol wash for 5 min and then with a 10% H_2_O_2_ wash for 2 h. The seeds were rinsed 5x with sterile ddH_2_O, plated on Luria-Bertani (LB) (Difco Laboratories, Detroit) agar plates, and incubated at 28 °C in humidity chambers (plastic boxes with moistened paper towels lining the bottom and the plates separated from the towels by glass petri plates). During the next 3–4 days, clean seeds were carefully separated from any seeds displaying signs of contamination and moved to fresh clean LB plates. After 6 days, clean germinated seeds were selected based on uniform root development and placed in hydroponic units. The hydroponic units consisted of wide mouth 16 oz. mason jars (Ball wide mouth canning jar 16 oz) with a 125 mL shaker clamp (Thermo Scientific™ MaxQ™ Shaker Universal Clamps, model 30,153) supporting a five-count plastic canvas mesh stage (circles cut from large plastic sheets and autoclaved separately prior to placing in sterile jars). The units were filled with approximately 225 ml of half strength Murashige and Skoog medium with Gamborg vitamins (pH = 5.6, Sigma-Aldrich, St. Louis, MO, U.S.A) amended with 0.05% sucrose. The tap root of each of five clean seedlings was threaded through the open squares in the plastic mesh insuring the ends of the roots were submerged in MS. The jars were capped with the bottom of sterile plastic 100 × 15 mm petri dishes, placed orbital shakers set at 50 rpm (New Brunswick) and incubated at 25–27 °C with a 16:8 light: dark photoperiod. After 5 days, 1 g *T. virens* mycelia was added to each jar through a notch cut into the mesh to facilitate adding the mycelia without contacting the roots. The mycelia were filtered from 24-h potato dextrose broth (PDB) cultures (1 L in Fernbach flasks) that had been inoculated with conidia (1 × 10 ^5^/ ml) from 10-day old colonies of *T. virens.* Mycelia were washed 5X with dH_2_O to remove traces of PDB media. Once the mycelia were added to a jar, a second mason jar (24 oz. wide mouth) was placed on top of the 16 oz. jar with parafilm wrapped at the point of contact. This arrangement allowed for unimpeded shoot growth.

### Harvesting of plants

Shoot tissue, root tissue, and fungal biomass were harvested after 6 and 30 h of co-cultivation. At 6 h of fungal: maize co-cultivation *T. virens* was visibly growing towards maize roots. Cultivation of maize roots indicated that *T. virens* had not yet colonized the maize roots (Additional file 4: Figure S2). Additionally, *T. virens* and maize were harvested separately for RNA extraction. We defined this timepoint as the “Recognition” phase. At 30 h of co-cultivation, the fungal and maize RNA were extracted from the same tissue sample as fungus was inextricable from maize at this timepoint. We defined this as the “Colonization” phase. After removal from hydroponic media all tissues samples were immediately stored in liquid nitrogen until preservation at − 80 °C.

### RNA extraction

RNA was extracted from ten biological replicates of maize co-cultivated with *T. virens* and four biological replicates of *T. virens* grown alone using a modified method for the Qiagen RNeasy Plant Mini Kit. The tissue samples were first ground in liquid nitrogen, and 100 mg of the tissue was aliquoted for RNA extraction. While the samples were still chilled in liquid nitrogen, 1 mL TRI reagent (Molecular Research Center Inc., Cat. TR118) was added to each sample and vortexed. The samples were then left at room temperature for 5 min before 200 μL chloroform was added and mixed by inversion. The samples incubated at room temperature for 10 min before being centrifuged at 13,000×g at 4 °C for 15 min. The supernatant was transferred to a new tube with 500 μL isopropanol. The samples were gently mixed by inversion and stored at room temperature for 10 min. The samples were then transferred into the Qiagen RNeasy Plant Mini Kit (Qiagen, Hilden, Germany, Cat# 74903) spin columns (pink). The samples were then processed following the remaining steps of the Qiagen RNeasy Plant Mini Kit’s instructions.

### RNA-sequencing

Total extracted RNA was provided to the Texas A&M AgriLifeGenomics and Bioinformatics Service. For samples extracted 30 h after fungal-maize co-cultivation, the submitted gRNA was composed of both fungal and plant RNA. Preliminary sequencing showed that this combined RNA sample was primarily fungal RNA. Samples extracted at 6 h were recombined in a 20:80 (fungal: maize) ratio to mimic the 30 h samples and allow sufficient material for sequencing of maize RNA; analysis of maize RNA will be presented elsewhere. cDNA libraries were created with the NEXTflex® Rapid Illumina Directional RNA-Seq Library Prep Kit. Sequencing was done on a NovaSeq 6000, for 50 bp paired end reads to a depth of 250 million reads.

### Data analysis

Base-calling, quality checking, and removal of adaptor sequences was performed by at the Texas A&M AgriLife Genomic and Bioinformatics Service as per their standard operating procedure. Raw, paired end, 50 bp reads were then aligned back to the *Trichoderma virens* Gv29–8 genome (NCBI accession number GCA_000170995.2) via the TopHat2 v2.1.0 pipeline [89] Alignment rates varied, dependent on whether samples were composed of only fungal RNA or a mix of fungal/plant RNA (Additional file 6: Figure S3). Uniquely aligned reads were counted with the HT-Seq 0.6.1 pipeline [90] using the GenBank GCA_000170995.2_TRIVI_v2.0_genomic file for annotation. Multi-dimensional scaling of rlog transformed HT-Seq data showed clear separation of fungal transcriptome clusters between treatments (Additional file 7: Figure S4).

FPKM values were calculated from the HT-seq output by the following equation:

$$ \left(\mathrm{read}\ \mathrm{number}/\mathrm{mRNA}\ \mathrm{kb}\right)/\left(\mathrm{total}\ \mathrm{number}\ \mathrm{of}\ \mathrm{reads}/{10}^6\right) $$

Differential expression analysis was conducted using the DeSeq2 1.16.1 pipeline which normalizes libraries based on the geometric mean of the read counts and then calculates the log_2_fold change between a defined ‘experimental’ and ‘reference’ sample [91]. Experimental samples were *T. virens* grown with maize and reference samples *T. virens* grown alone. DEGs (differentially expressed genes) were considered significant if a log_2_fold change was ±1.5 with a p adjusted value of < 0.05. Significant DEGs were manually assigned general categories of function based on the Kyto Orthology of Genes (KEGG, [92]), GO Term (Gene Ontology, [93]), CAZY (Carbohydrate-Active enZymes Database, [94]), and protein domains as annotated in JGI (Joint Genome Institute, [95]) version 2.0 *T. virens* Gv29–8 catalogue [31]. These functional categories are not meant to represent absolute function, but a general method of data organization.

### RNA-sequencing validation

Gene expression identified by RNA-seq was validated with quantitative reverse-transcriptase polymerase chain reaction (RT-qPCR). Validation genes were selected to represent a range of log_2_fold changes, between the fungus grown alone and with maize at both the Recognition and Colonization datasets (Additional file 8: Table S1). RNA was freshly extracted from three biological replicates of plant and fungal tissue from each condition which had been stored at -80ͦC. These frozen tissue samples were collected in the original hydroponics experiment but were replicates not submitted for RNA-Seq. RNA was extracted as described above (section, *RNA Extraction.*). 2 μg of total RNA in a 20 μl reaction was converted to cDNA with a High Capacity cDNA Reverse Transcription Kit (Applied Biosystems, USA) by manufacturer operating procedure on a BLANK thermocycler with the following conditions: 25 °C for 10 min, 37 °C for 120 min, 85 °C for five minutes, followed by a hold at 4 °C until use in RT-qPCR reaction. 1 μl of cDNA reaction was used as the input for qPCR reaction. qPCR reactions were made with a PowerUp SYBR Green Kit (Applied Biosystems, USA) by manufacturer operating procedure in a 10 μl volume. qPCR was done a StepOne Plus Real-Time PCR system with the following cycling conditions: 50 °C for 2 min, 95 °C for 2 min, followed by 40 cycles of 95 °C (15 s), 53 °C (15 s), 72 °C (15 s). Melt curve conditions were 95 °C for 15 s, 60 °C for 1 min, 95 °C for 15 s. All samples had only one melt temperature peak. Log_2_fold change between experimental samples (*T. virens* with maize) and control samples (*T. virens* grown alone) was calculated by the 2^^-Δ ΔCT^ method using actin as a reference gene (Additional file 8: Table S1). CT values represent the average of three technical replicates (Additional file 1: Figure S1).

### Identification of secondary metabolite clusters

Sequences of the genes in the vicinity of signature genes (e.g., NRPS, PKS, terpene cyclase) in respective scaffolds were analyzed for presence of domains putatively involved in secondary metabolism (like cytochrome P450, oxidoreductase, glutathione S-transferase) by NCBI CDD database search. The boundaries of the putative gene clusters were defined by the presence of genes not known to be involved in secondary metabolism.

## Additional files

Additional file 1: **Figure S2.** *T. virens* has recognized but not colonized B73 Maize after 6 h of hydroponic co-cultivation. Plant grow hydroponically, as described in methods, were harvested at 6 then 30 h post inoculation with *T. virens* (*N* = 3 per harvest). Roots were dissected into 1 cm pieces and plated on GVSM. The number of root pieces with fungal growth after three days of incubation at 27 °C was counted and divided by the total root pieces plates to get a percentage colonized for each plant. (PPTX 35 kb)

Additional file 2: **Data file S1.** Excel file with log_2_fold changes and p-adjust values for all genes in the *T. virens* Gv29–8 genome at Recognition and Colonization and calculated FPKM values. (XLSX 5288 kb)

Additional file 3: **Data file S2.** Excel file listing the log_2_fold changes and p-adjust values for all significant DEGs at Recognition and Colonization, with assigned category and function notes. (XLSX 95 kb)

Additional file 4: **Figure S1.** RT-qPCR validation of RNA-Seq based differential expression analysis. 9 DEGs representing a range of log2fold changes and expression in the Recognition vs Colonization datasets were chosen for validation with RT-qPCR (total of 18 data-points). Points are colored by DEG ID # to highlight RT-qPCR still captures the direction of the log2fold change when DEGs were oppositely regulated between Recognition and Colonization. The direction of the log_2_fold change was in agreement between the two methodologies, though the magnitude of said change was different. This is explicable by the different kits used for cDNA conversion and that the RT-qPCR samples were extracted from tissue held at -80ͦC for 12 months whereas samples used in the RNA-Seq analysis were extracted within one month. Pearson correlation was 0.75. (PPTX 38 kb)

Additional file 5: **Data file S3.** Excel file containing database of all genes identified as belonging to a secondary metabolite gene cluster. (XLSX 31 kb)

Additional file 6: **Figure S3.** Percentage of reads aligning to the *T. virens* genome and the sequenced coverage of the *T. virens* represented by aligned reads. (PPTX 43 kb)

Additional file 7: **Figure S4.** Multi-dimensional plot of each RNA-seq library: *T. virens* grown without maize at 6 h (no B73_6hr), *T. virens* growth without maize at 30 h (no B73_30hr), *T. virens* cultivated with maize for 6 h (with B73_6hr) and for 30 h (with B73_30hr). Clustering shows close intra-condition clustering. (PPTX 117 kb)

Additional file 8: **Table S1.** List of genes and primer sequences used for RT-qPCR validation of RNA-Seq differential expression analysis. (DOCX 14 kb)

## Abbreviations

CAZ: Carbohydrate activated enzyme
CWDE: Cell wall degrading enzyme
DE: Differential expression
DEG: Differentially expressed gene
IAA: Indole-3-acetic acid
JA: Jasmonic acid
RT-qPCR: Quantitative-real-time polymerase chain reaction
SA: Salicylic acid
