# Supplementary figures and images for: Differential expression analysis of Trichoderma virens RNA reveals a dynamic transcriptome during colonization of Zea mays roots
Source: BMC Genomics. 2019 Apr 11;20:280. doi: 10.1186/s12864-019-5651-z (PMC6458689; doi:10.1186/s12864-019-5651-z)

## Slide 1
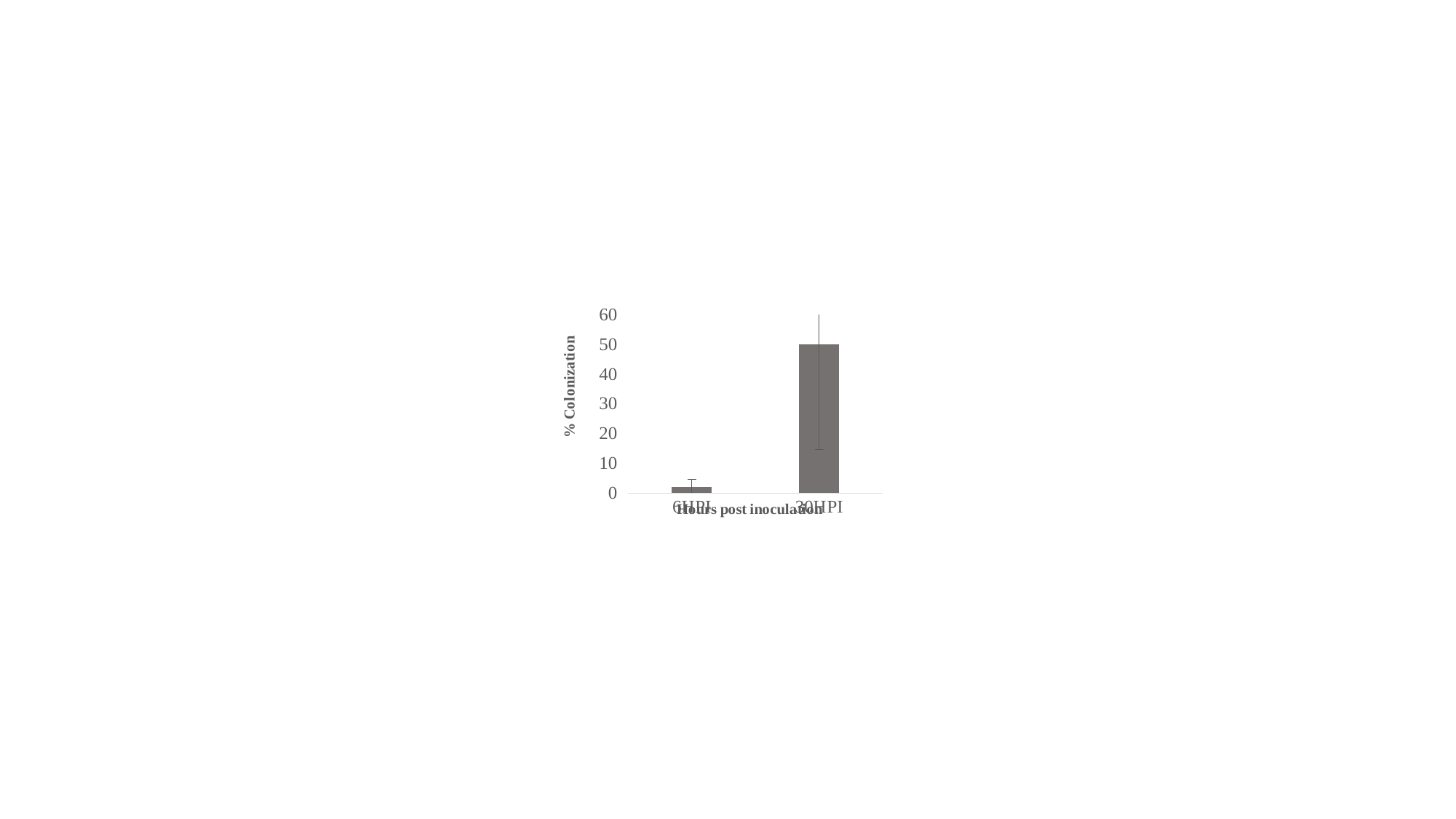

### Chart
| Category | |
|---|---|
| 6HPI | 2.0833333333333335 |
| 30HPI | 50.0 |

Supplement: Supplementary file 1 — Figure S2. T. virens has recognized but not colonized B73 Maize after 6 h of hydroponic co-cultivation. Plant grow hydroponically, as described in methods, were harvested at 6 then 30 h post inoculation with T. virens (N = 3 per harvest). Roots were dissected into 1 cm pieces and plated on GVSM. The number of root pieces with fungal growth after three days of incubation at 27 °C was counted and divided by the total root pieces plates to get a percentage colonized for each plant. (PPTX 35 kb) [file 12864_2019_5651_MOESM1_ESM.pptx]

## Slide 1
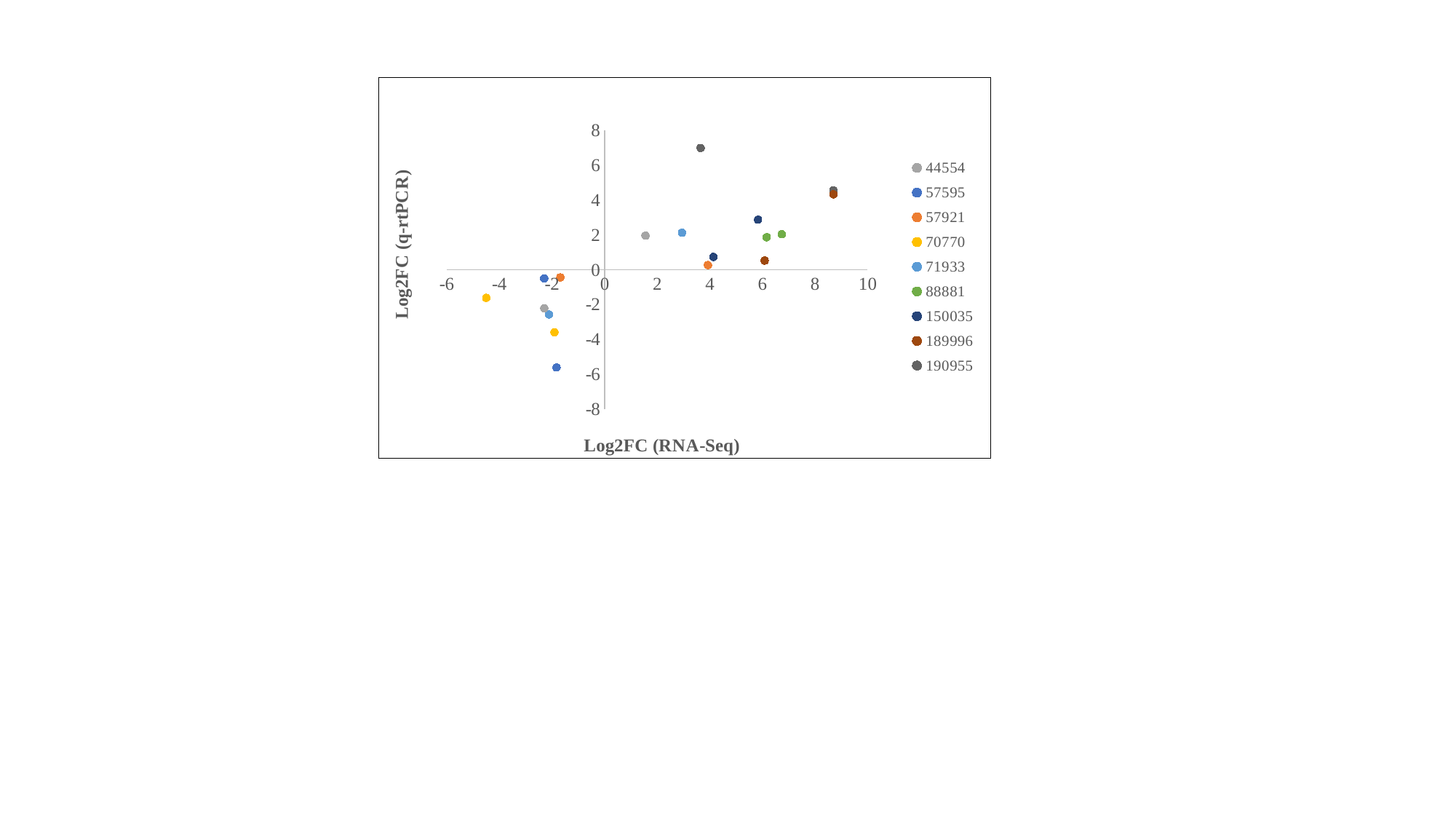

### Chart
| Category | 44554 | 57595 | 57921 | 70770 | 71933 | 88881 | 150035 | 189996 | 190955 |
|---|---|---|---|---|---|---|---|---|---|

Supplement: Supplementary file 4 — Figure S1. RT-qPCR validation of RNA-Seq based differential expression analysis. 9 DEGs representing a range of log2fold changes and expression in the Recognition vs Colonization datasets were chosen for validation with RT-qPCR (total of 18 data-points). Points are colored by DEG ID # to highlight RT-qPCR still captures the direction of the log2fold change when DEGs were oppositely regulated between Recognition and Colonization. The direction of the log2fold change was in agreement between the two methodologies, though the magnitude of said change was different. This is explicable by the different kits used for cDNA conversion and that the RT-qPCR samples were extracted from tissue held at -80ͦC for 12 months whereas samples used in the RNA-Seq analysis were extracted within one month. Pearson correlation was 0.75. (PPTX 38 kb) [file 12864_2019_5651_MOESM4_ESM.pptx]

## Slide 1
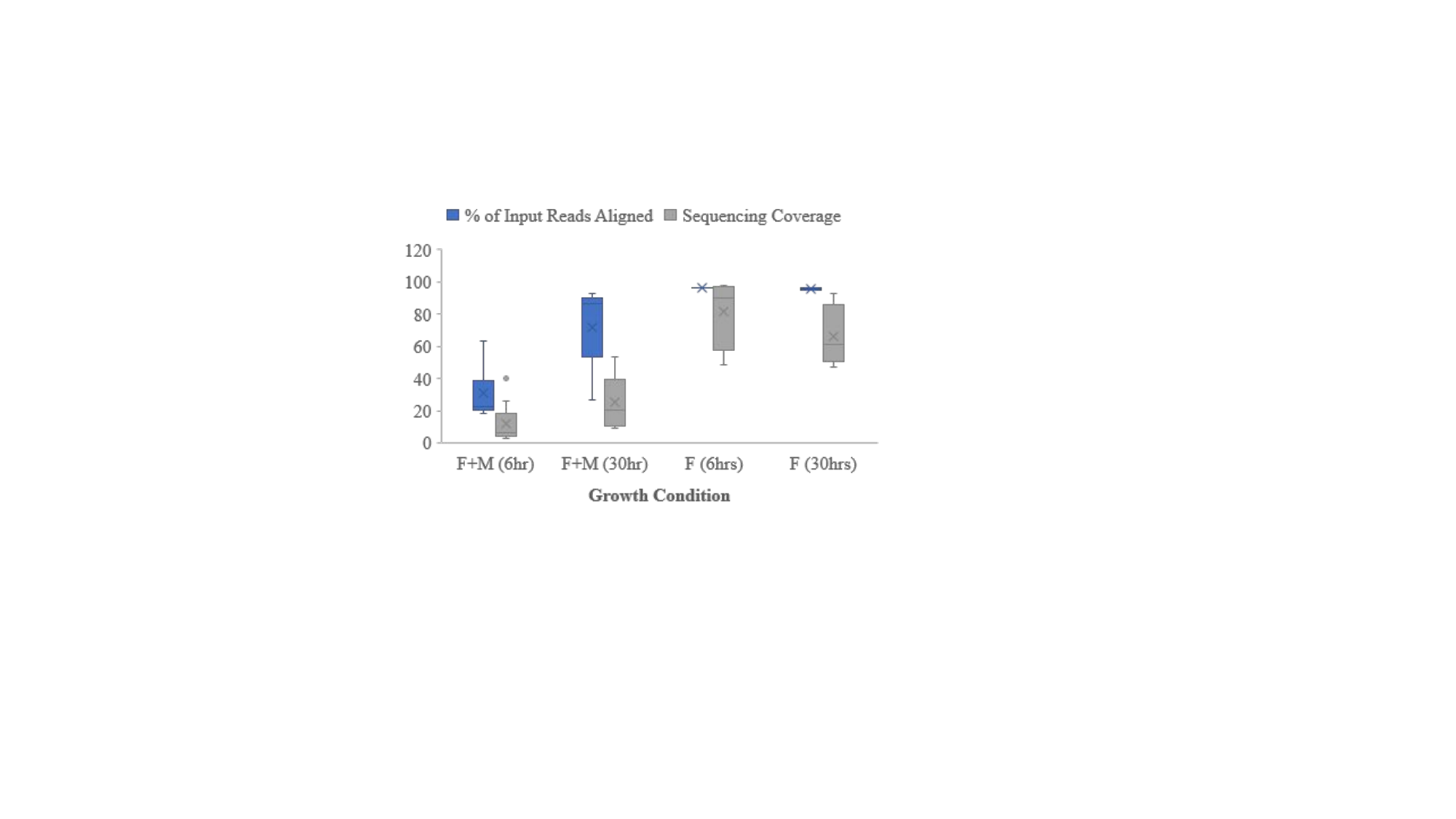

Supplement: Supplementary file 6 — Figure S3. Percentage of reads aligning to the T. virens genome and the sequenced coverage of the T. virens represented by aligned reads. (PPTX 43 kb) [file 12864_2019_5651_MOESM6_ESM.pptx]

## Slide 1
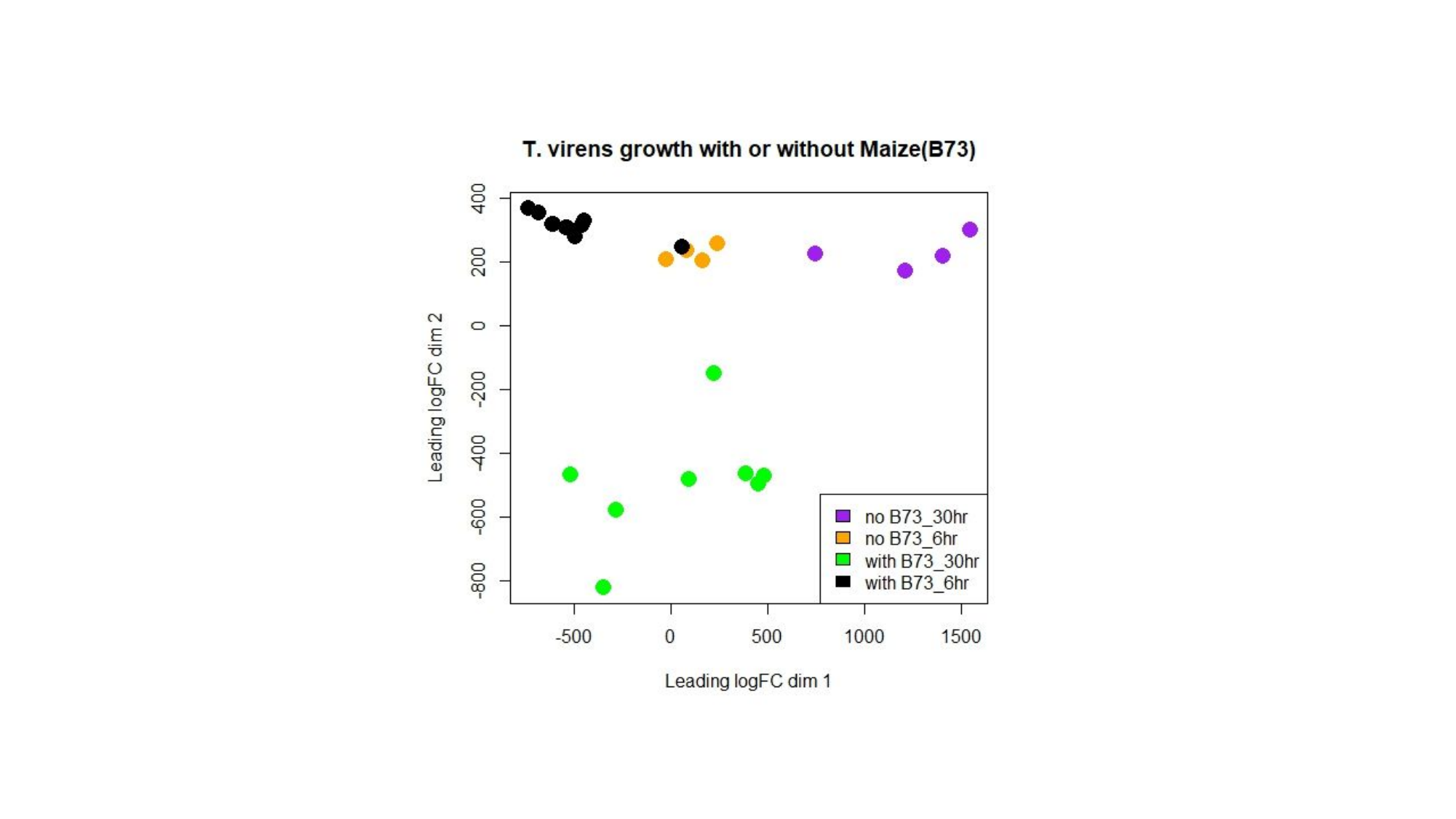

Supplement: Supplementary file 7 — Figure S4. Multi-dimensional plot of each RNA-seq library: T. virens grown without maize at 6 h (no B73_6hr), T. virens growth without maize at 30 h (no B73_30hr), T. virens cultivated with maize for 6 h (with B73_6hr) and for 30 h (with B73_30hr). Clustering shows close intra-condition clustering. (PPTX 117 kb) [file 12864_2019_5651_MOESM7_ESM.pptx]
