# Supplementary material for: Differential expression analysis of Trichoderma virens RNA reveals a dynamic transcriptome during colonization of Zea mays roots
Source: BMC Genomics. 2019 Apr 11;20:280. doi: 10.1186/s12864-019-5651-z (PMC6458689; doi:10.1186/s12864-019-5651-z)
Supplement: Supplementary file 8 — Table S1. List of genes and primer sequences used for RT-qPCR validation of RNA-Seq differential expression analysis. (DOCX 14 kb) [file 12864_2019_5651_MOESM8_ESM.docx]

**Supplemental Table 1.** List of genes and primer sequences used for q-rtPCR validation of RNA-Seq differential expression analysis.

| **Gene ID; Function** | **Forward Primer (5’ – 3’)** | **Reverse Primer (5’ – 3’)** |
| --- | --- | --- |
| Actin; 185456* | GTATCATGATCGGTATGGGTCAGA | TAGAAGGTGTGGTGCCAGATCTT |
| 135160; C-type lectin | ATACTTGCGACGATCCCGAC | GCTGGTGTGGAAGTTGCTTG |
| 190955; Cytochrome P450 | TCAATACCGCTTTACGCCGA | CATTGTCCCAGAGCTCAGCA |
| 44554; Amino acid transporter | CTCTCCAGGCGGTCTTTCTG | GAGTGCACCCGTCAACGTAT |
| 71933; Trypsin | CTTCTGCGGTGGTGTTTTGG | ATGGTGGAGCTCTGAGGGAT |
| 57921; MFS transporter | CTCGTGCTGGTACAAGCGTA | AGGAATTTGGCCTCATCGGG |
| 66683; Chitinase | CACGGCATCATTGGCTTCAG | CGACCGTTGGGTTCAAGGTA |
| 57595; Kynurenine 3-monooxygenase | TTGTTATCGGCGCTGATGGT | GTCGCCCATTTGCTGAAGAG |
| 61018; NADP/FAD dep. oxidoreductase | CATGCGAGGCTTTGGCATAC | AGGAGTCCTTTTCAGCCGTG |
| 88881; Catalasae | GCGCTGACCCTAACTACCTC | TGGCTCCAGACAAATGACCC |
| 216161; Cytochrome P450 | CAACAGCGGTTGGCTTATCG | TGGAAACATCTTCAGCGCCT |
| 47897; Glutathione S-transferase | GTTTGAGCATAAGCGCCCAG | ACGGCGTCGAAGGATGTAAG |
| 70770; NRSP | CAAAGTCATGATGCCGAGCG | CGGAGCTATTCATCGGCAGT |
| 57245; carbon-nitrogen hydrolase | ATCCCCGATCGGCAACATTT | CCAGTCTCGTGGGCAATTCT |

* housekeeping gene used for q-rtPCR normalization.
